# Prediction of sediment transport capacity based on slope gradients and flow discharge

**DOI:** 10.1371/journal.pone.0256827

**Published:** 2021-09-07

**Authors:** Kai Zhang, Wang Xuan, Bai Yikui, Xu Xiuquan

**Affiliations:** 1 College of Water Conservancy Shenyang Agricultural University, Shenyang City, Liaoning Province, P.R. China; 2 Laboratory of Soil Erosion Control and Ecological Restoration in Liaoning Province, Shenyang City, Liaoning Province, P.R. China; Ton Duc Thang University, VIET NAM

## Abstract

Sediment transport capacity (*T*_*c*_) is an essential parameter in the establishment of the slope soil erosion model. Slope type is an important crucial factor affecting sediment transport capacity of overland flow, and vegetation can effectively inhibit soil loss. Two new formulae of sediment transport capacity (*T*_*c*_) are proposed of brown soil slope and vegetation slope in this study and evaluate the influence of slope gradient (*S*) and flow discharge (*Q*) on sediment transport capacity of different slope types. Laboratory experiments conducted using four flow discharges (0.35, 0.45, 0.55, and 0.65 L s^-1^), four slope gradients (3, 6, 9, and 12°), and two kinds of underlying surface (Brown soil slope, Vegetation slope). The soil particle size range is 0.05–0.5mm. The vegetation stems were 2mm in diameter and randomly arranged. The results show that the sediment transport capacity was positively correlated with the flow discharge and slope gradient. The vegetation slope’s average sediment transport capacity is 11.80% higher than the brown soil slope that same discharge and slope gradient conditions. The sensitivity of sediment transport capacity to flow discharge on brown soil slope is higher than that of slope gradient. The sensitivity of sediment transport capacity of vegetation slope to slope gradient is more heightened than flow discharge. The sediment transport capacity was well predicted by discharge and slope gradient on brown soil slope (R^2^ = 0.982) and vegetation slope (R^2^ = 0.993). This method is helpful to promote the study of the sediment transport process on overland flow.

## 1. Introduction

Sediment transport capacity is one of the most critical factors influencing soil erosion [1, 2]. It significantly affects soil erosion’s movement process [3, 4]. Sediment transport capacity is a comprehensive index when the sediment deposition and transport balance. Furthermore, it is imperative to study sediment yield, sediment transport, and sediment deposition.

Many scholars use the concept of sediment transport rate to research the sediment transport capacity of overland flow. The commonly used unit is kg (m·s)^-1^, and it is a product of sediment concentration and unit discharge [4]. To eliminate the influence of unit discharge, adopted the sediment concentration concept in this study. Under particular flow and boundary conditions, maximum sediment concentration was carried by unit flow energy, and the unit is kg m^-3^. Several scholars have carried out many experimental studies, and they found that the sediment transport capacity was significant affected by the slope gradients and flow discharge [2, 5]. Some scholars also use artificial intelligence (AI) to simulate sediment transport [6–8]. Based on the developed integrated machine learning models, it is found that the discharge has an essential effect on the suspended sediment concentration [7]. However, this method needs a lot of high-quality experimental data as the basis [8]. Different reference factors are independent variables in exploring the equation for sediment transport capacity on overland flow. The main variables affecting sediment transport capacity are slope gradients and flow discharge [9].

Beasley [10] concluded that the relationship between sediment transport capacity with slope gradients and flow discharge that the data of sediment transport capacity of overland flow are summarized and analyzed:

$$T_{c}=146q^{0.5}S\;q\leq 0.046$$ (1)

$$T_{c}=14600q^{2}S\;q>0.046$$ (2)

Where *T*_*c*_ is sediment transport capacity, kg·(m·min)^-1^; *q* is flow discharge, m^2^·min^-1^; *S* is slope gradients, %. It represents the power function relation of sediment transport capacity with slope gradients and flows discharge. However, due to the different experimental conditions, the results obtained are slightly different. For example, Soil types can affect the sediment transport capacity of overland flow [11, 12]. Furthermore, vegetation plays a vital role in process prevention and cures to soil erosion [13]. Compared with the bare slope, vegetation slope has a higher surface roughness [14]. It increases the flow resistance of sediment-laden flow, reduces the flow velocity [12] and sediment transport capacity of water flow [15, 16].

In summary, research on sediment transport to simulate runoff by slope and discharge is an effective method [17]. The northeast black soil area in China has a slight slope and high rainfall intensity. The previously established equations for predicting sediment transport capacity have different slope gradients and flow discharge conditions. Seldom studies have unequivocal sediment transport capacity in brown soil and vegetation on slopes. Therefore, in this study, vegetation slope and brown soil slope were taken as the research objects, and the scour tests with different slope gradients and flow discharge combinations were carried out. Two new prediction model for sediment transport capacity of brown soil and vegetation slope was proposed. This study can provide data for artificial intelligence simulation of sediment transport, helpful for developing a soil erosion process model of the black soil region of Northeast China that provides a theoretical basis.

## 2. Materials and methods

### 2.1 Study area and test soil

The experimental soil samples were collected from Shenyang Agricultural University Water Conservancy Institute Comprehensive Experimental Base (123°27’ E, 41°44’ N, with an altitude of 44.7 m). The soil sample belongs to brown soil, and before the test, it needs not only to be air-dried and filtered by a sieve with sieve pores 10 mm on each edge, through which the contaminants (such as plants roots, rubbles, and other debris) can be removed but also identified its particle size by sieving method. The soil particles were mainly medium sand (0.25–0.5 mm) and fine sand (0.05–0.25 mm), accounting for 45.26% and 41.27%, respectively, as shown in Table 1. The average soil organic matter content was 21.8 g·kg^-1^, the soil moisture content was 15%, the pH value of 6.62, and the median particle size d_50_ of the test soil is 0.29 mm. The soil was uniformly bonded to the flume’s surface to simulate the natural underlying surface’s roughness.

**Table 1:** The distribution of particle sizes.

**Particle size (mm)**	<0.001	0.001~0.005	0.005~0.01	0.01~0.05	0.05~0.25	0.25~0.5	0.5~1.0	1.0~2.0
**Percentage (%)**	0.88	1.12	1.48	4.82	41.27	45.26	3.68	1.49

### 2.2 Experiment facilities

**Fig 1:**
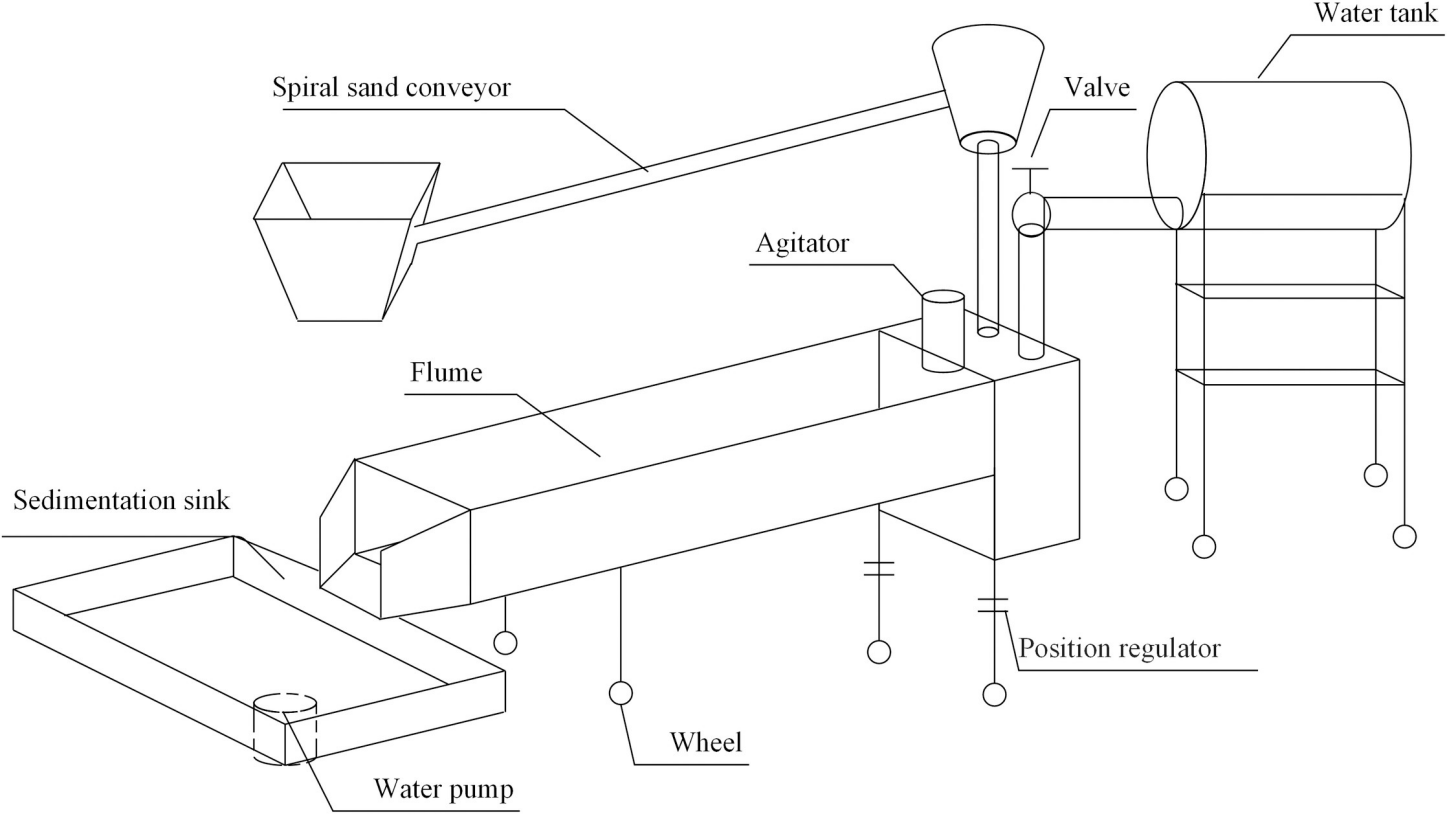
Schematic diagram of the test device.

**Fig 2:**
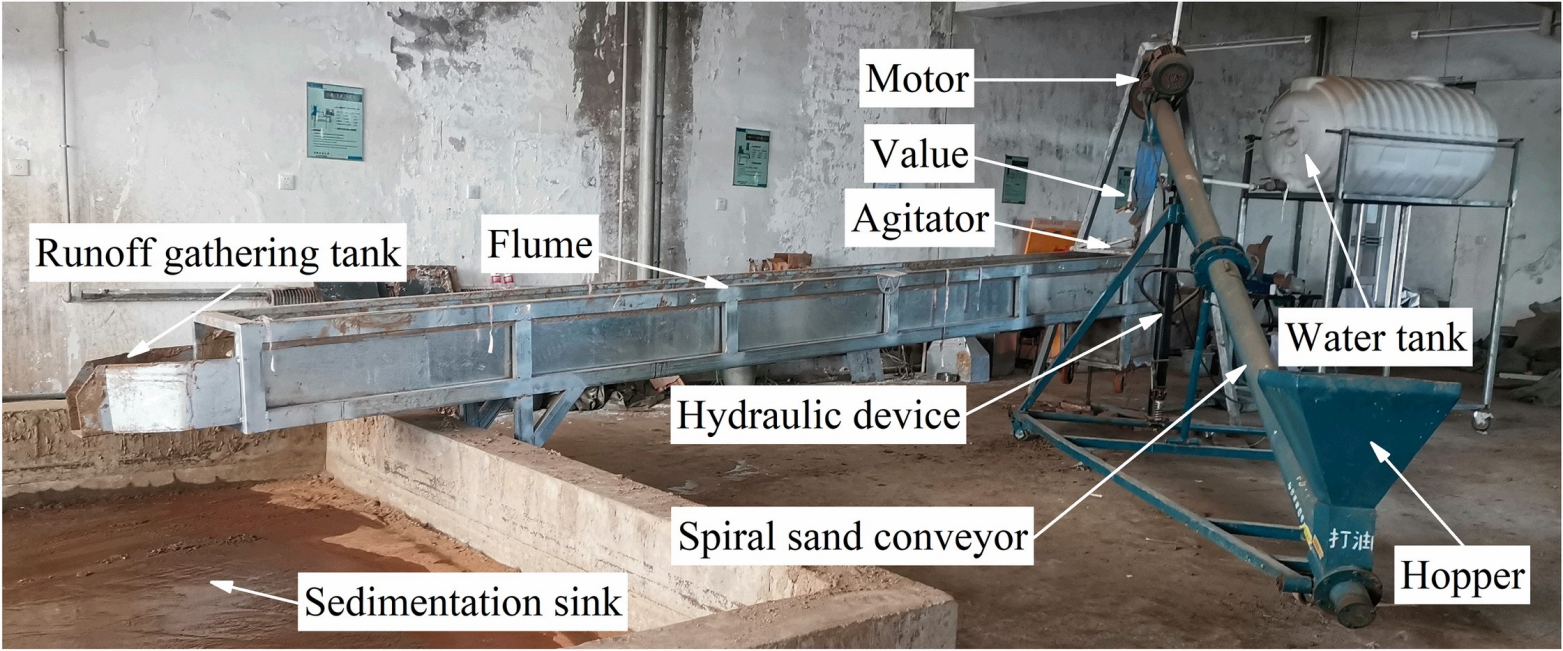
Experiment facilities.

The study was carried out in the Hydraulic Hall of the Water Conservancy Institute, Shenyang Agricultural University. The experiment facilities mainly include the water tank, flume, spiral sand conveyor, agitator, and sedimentation sink (Figs 1 and 2). The experimental flume used in this study was about 6 m long, 0.43 m wide, and a height of 0.4m. The slope gradients of the flume could be adjusted between 0° and 15° of the lifting pulley. The soil was uniformly adhered to the flume surface by paints [9]. After air drying, the brown soil slope scouring experiment was carried out. Based on the brown soil slope arrangement, the vegetation (Helictotrichon schellianum) was placed on the painted flume, and a layer of soil is added evenly [12]. The stems adhered to the flume bed, the stems were 2mm in diameter, and 30% of the branches were randomly arranged, and the canopy coverage was nearly 100%. A small water tank of 0.5m×0.43m×0.8m (length × width × height) is set at the upper end of the flume. At the end of the flume, a runoff gathering tank with a width of 0.15 m was set.

Water is supplied to the capacity water tank by the water pump, and the volume of the water tank is 1t. Flow discharge was controlled by a set of valves installed on a flow diversion bin and measured by a calibrated flow meter. Moreover, it was directly measured three times by a calibrated flow-meter, and the average value is the flow discharge [2]. Control of sediment load (0.464 kg·s^-1^) in the water at the flume inlet by spiral sand conveyor. Agitators mix water and sediment evenly. The sedimentation sink at the flume bottom collects runoff and sediment from the slope.

A hydraulic device can adjust the height of the spiral sand conveyor. The screw conveyor was installed between the sediment hopper and the sediment outlet, connected to the motor through the belt [9, 12]. The regulator adjusts the operating speed of the motor and then controls the speed of the conveyor. At the same time, a movable switch was installed at the bottom of the hopper. The speed of Gaza was controlled by a combination of transmissions device and movement switches [2, 12].

The agitator was fixed in the small water sink, mix the water and sediment homogeneous, insert a 0.43 m wide × 0.15 m high baffle in the small water tank and the flume joint, and let the silt carrying flow evenly and steadily into the flume. The runoff gathering tank was used to collect water and sediment.

### 2.3 Experimental design

Based on field observations, the slope of arable land is generally 0° - 15° in the Northeast. The average annual precipitation in Northeast China is 600–800 mm. Generally, the frequency of hefty rain in 30 to 180 min time frames was the highest. The unit flow discharge rate for the test rain intensity was set between 30 and 120 mm h^-1^ based on the local confluence area [18]. Therefore, the test was performed by adopting different combinations of four slope gradients (3, 6, 9, 12°), four flow discharges (0.35, 0.45, 0.55, 0.65L s^-1^), and two kinds of underlying surface (Brown soil slope, Vegetation slope), as shown in Table 2. So there are 32 different combinations in total, and each combination was repeated four times, and a total of 128 times.

**Table 2:** Design of experiment.

slope gradients /(°)	Flow discharges /(L s^-1^)			
3	0.35	0.45	0.55	0.65
6	0.35	0.45	0.55	0.65
9	0.35	0.45	0.55	0.65
12	0.35	0.45	0.55	0.65

### 2.4 Experimental process

Calibrate and check the spiral sand conveyor before each trial, and control the flow discharges and slope gradients to reach the design value. When the flow stabilized, it started in Gaza. The timing will start when the sediment comes to a critical siltation state at the runoff gathering tank, and sediment transport capacity was assumed to be reached [19, 20]. In order to prevent excessive siltation on the flume surface, samples were taken every 10 s, and a total of 6 water and soil mixed samples were collected in each test. The middle four groups of sample data were selected for the calculation to minimize the error. Measuring the water-soil mixed sample volume with a measuring cylinder, and the sum of the volume of 4 samples is the water-soil mixed sample volume (*V*). All samples from each experiment were allowed to settle for 24 hours. The supernatant was discarded, and the wet sediments were oven-dried at 105°C for 12 hours. Use a balance to weigh the dried soil sample, and the sum of 4 sediment samples in each test is the dry weight of sediment (*M*). The tests were four repeated experiments. The data set of this experiment was divided into two parts: three groups of experimental data were used to establish the formula of sediment transport capacity, and one group of test data was used to validate the formula. The experimental data results were shown in Table 3.

**Table 3:** Experimental results.

Slope gradients (°)	Flow discharges (L s^-1^)	Three groups average sediment transport capacity /(kg m^-3^)		One group sediment transport capacity /(kg m^-3^)	
		Brown soil	Vegetation	Brown soil	Vegetation
3	0.35	374.86	306.03	378.45	320.70
3	0.45	405.06	327.54	393.06	321.18
3	0.55	412.60	342.98	414.16	335.55
3	0.65	428.51	359.00	430.41	358.23
6	0.35	406.59	373.10	417.30	375.00
6	0.45	434.50	390.80	435.93	389.08
6	0.55	451.81	400.60	445.09	395.16
6	0.65	471.11	409.02	481.23	411.24
9	0.35	432.96	405.26	427.17	401.26
9	0.45	473.19	423.40	468.89	435.21
9	0.55	495.55	441.82	482.02	444.72
9	0.65	502.26	453.24	513.44	453.72
12	0.35	454.38	437.96	461.71	426.48
12	0.45	492.66	459.05	491.47	455.37
12	0.55	511.59	479.19	492.79	466.88
12	0.65	530.35	501.42	525.45	489.00

### 2.5 Statistical analysis

All statistical analyses were carried out using R studio. The average values of three groups of repeated experiments were used to derive the equations of the relationship between sediment transport capacity and slope gradient and flow discharge on brown soil and vegetation slopes. The relative error (RE), the mean relative error (MRE), the mean absolute relative error (MARE), the determination coefficient (R^2^), the Nash-Sutcliffe model efficiency (NSE) were used to evaluate the simulated results of the equations [17]. They reflect the reliability and precision of the model.

## 3. Results

### 3.1 Relationship between sediment transport capacity with slope gradient and flow discharge

**Fig 3:**
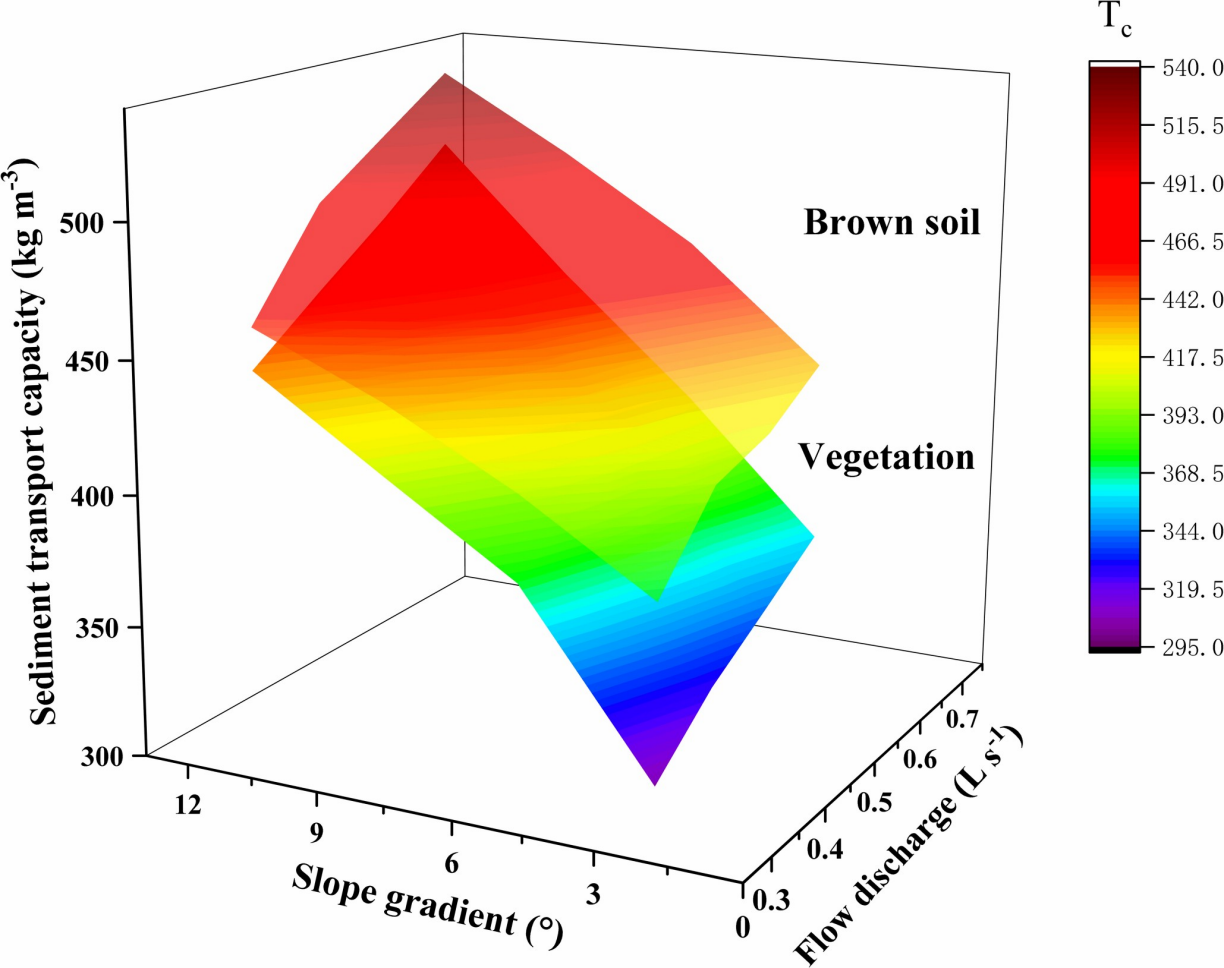
The sediment transport capacity changing the relationship with slope gradients and flow discharge.

Slope gradients and flow discharge are essential factors in sediment transport, and they are the main factors to determine the overland’s sediment transport capacity. Therefore, the sediment transport capacity changing the relationship has drawn into the three-dimensional mapping surface with slope gradients and flow discharge (Fig 3).

As shown in Fig 3, the sediment transport capacity increased with increasing slope gradients and flow discharge within this test range. The sediment transport capacity variation ranged was between 374.86 kg·m^-3^ ~ 530.35 kg·m^-3^ for the brown soil slope, and it was between 306.03 kg·m^-3^ ~ 501.42 kg·m^-3^ for the vegetation slope. The average sediment transport capacity of the brown soil slope is 11.80% higher than that of the vegetation slope. This result suggests that with the increase of slope gradient and flow discharge, the kinetic energy and potential energy of sediment carried by the flow gradually increase [9, 17]. As the roughness of the vegetation slope is more significant than that of the brown soil slope, the vegetation inhibits sediment transport, resulting in the overall sediment transport capacity of the vegetation slope is less than that of the brown soil slope [12]. The sediment transport capacity increased with increasing flow discharge when the slope gradients were the same. The average increase in the brown soil slope’s sediment transport capacity was 15.79% when the flow discharge increased from 0.35 L·s^-1^ to 0.65 L·s^-1^, and the vegetation slope was 13.16%. The sediment transport capacity increased with increasing slope gradients when the flow discharges were the same. The average increase in the brown soil slope’s sediment transport capacity was 22.70% when the slope gradients increased from 3° to 12°, and the vegetation slope was 40.59%. The sediment transport capacity increased by 41.48% for brown soil slope and increased by 63.85% for vegetation slope when the slope gradients and flow discharge increase together. It can be seen that the vegetation slope had a more significant influence on sediment transport capacity compared with the brown soil slope.

The equations between mean slope gradients, flow discharge, and sediment transport capacity, respectively, further analyze the response relationship. It shows in Table 4.

**Table 4:** Regression equation between slope gradients (*θ*), flow discharge (*Q*), and sediment transport capacity (*T*_*c*_), respectively.

Underlying surface			Regression equation	F	P	R^2^	AIC
Brown soil slope	Slope gradients (°)	3	*T*_*c*_ = 168.49*Q*+321.01	73.88	6.25×10^−6^	0.881	50.46
		6	*T*_*c*_ = 210.89*Q*+335.56	66.16	1.02×10^−5^	0.869	57.17
		9	*T*_*c*_ = 230.26*Q*+360.86	47.96	4.07×10^−5^	0.828	63.14
		12	*T*_*c*_ = 246.84*Q*+373.83	35.97	1.33×10^−4^	0.783	68.26
	Flow discharge (L s^-1^)	0.35	*T*_*c*_ = 8.83*θ*+350.97	99.37	1.64×10^−6^	0.909	57.77
		0.45	*T*_*c*_ = 10.05*θ*+357.97	321.5	6.23×10^−9^	0.97	46.78
		0.55	*T*_*c*_ = 11.357*θ*+382.712	52.75	2.72×10^−5^	0.841	71.4
		0.65	*T*_*c*_ = 11.221*θ*+398.9	385.7	2.56×10^−9^	0.975	47.24
Vegetation slope	Slope gradients(°)	3	*T*_*c*_ = 174.357*Q*+246.708	114.3	8.58×10^−7^	0.92	46.04
		6	*T*_*c*_ = 117.56*Q*+334.6	15.04	3.07×10^−3^	0.601	60.92
		9	*T*_*c*_ = 162.37*Q*+349.743	104.8	1.28×10^−6^	0.913	45.38
		12	*T*_*c*_ = 210.54*Q*+364.13	8.665	1.47×10^−2^	0.464	81.53
	Flow discharge (L s^-1^)	0.35	*T*_*c*_ = 14.265*θ*+273.6	209.4	4.93×10^−8^	0.954	60.33
		0.45	*T*_*c*_ = 14.238*θ*+293.41	274.8	2.20×10^−8^	0.961	58.27
		0.55	*T*_*c*_ = 14.995*θ*+303.688	194.1	7.10×10^−8^	0.951	62.44
		0.65	*T*_*c*_ = 15.716*θ*+312.798	42.38	6.81×10^−5^	0.809	81.83

Table 4 shows the regression equations showing an increasing linear trend for sediment transport capacity related to slope gradients and flow discharge. Under different slope gradient conditions, the flow discharge coefficient was between 168.49 and 246.84 for brown soil slope, and the average value was 214.12. The flow discharge coefficient was between 117.56 and 210.54 for the vegetation slope, and the mean value was 166.207. Similarly, under different flow discharges, the slope gradient conditions coefficient was between 8.83 and 11.357 for brown soil slope, and the mean value was 10.365. the slope gradient conditions coefficient was between 14.265 and 15.716 for the vegetation slope, and the mean value was 14.804. It shows that the flow discharges had a more significant influence on sediment transport capacity with slope conditions.

### 3.2 Estimation of sediment transport capacity

The data set of this experiment was divided into two parts. Part of it is the data of 3 repeated experiments in 4 repeated experiments, which was used to derive the relationship equation between sediment transport capacity (*T*_*c*_) with slope gradient and flow discharge of brown soil slope. The results are shown in Table 5.

**Table 5:** Results of stepwise regression analysis.

Underlying surface	Model	Factors	Estimate	Std. Error	P	t	F	R^2^	AIC
Brown soil slope	Linear equation		270.079	10.812	2.26×10^−12***^	24.98	198.1	0.968	71.09
		*θ*	10.365	0.632	4.59×10^−10***^	16.39			
		*Q*	214.119	18.965	4.33×10^−08***^	11.29			
	Power equation		6.002	0.017	< 2×10^−16 ***^	351.36	363.6	0.982	-133.98
		*θ*	0.149	0.007	1.05×10^−11 ***^	22.14			
		*Q*	0.233	0.015	9.96×10^−10 ***^	15.40			
Vegetation slope	Linear equation		212.771	10.093	1.95×10^−11 ***^	21.08	358.7	0.982	68.89
		*θ*	14.803	0.59	2.14×10^−12 ***^	25.08			
		*Q*	166.206	17.705	3.73×10^−07***^	9.38			
	Power equation		5.685	0.015	< 2×10^−16 ***^	373.7	935.1	0.993	-137.69
		*θ*	0.243	0.006	4.43×10^−15 ***^	40.59			
		*Q*	0.201	0.013	1.48×10^−09 ***^	14.91			

Notes: *t*_0.01_13 = 3.012; F_0.01_(2,13) = 6.701.

**Fig 4:**
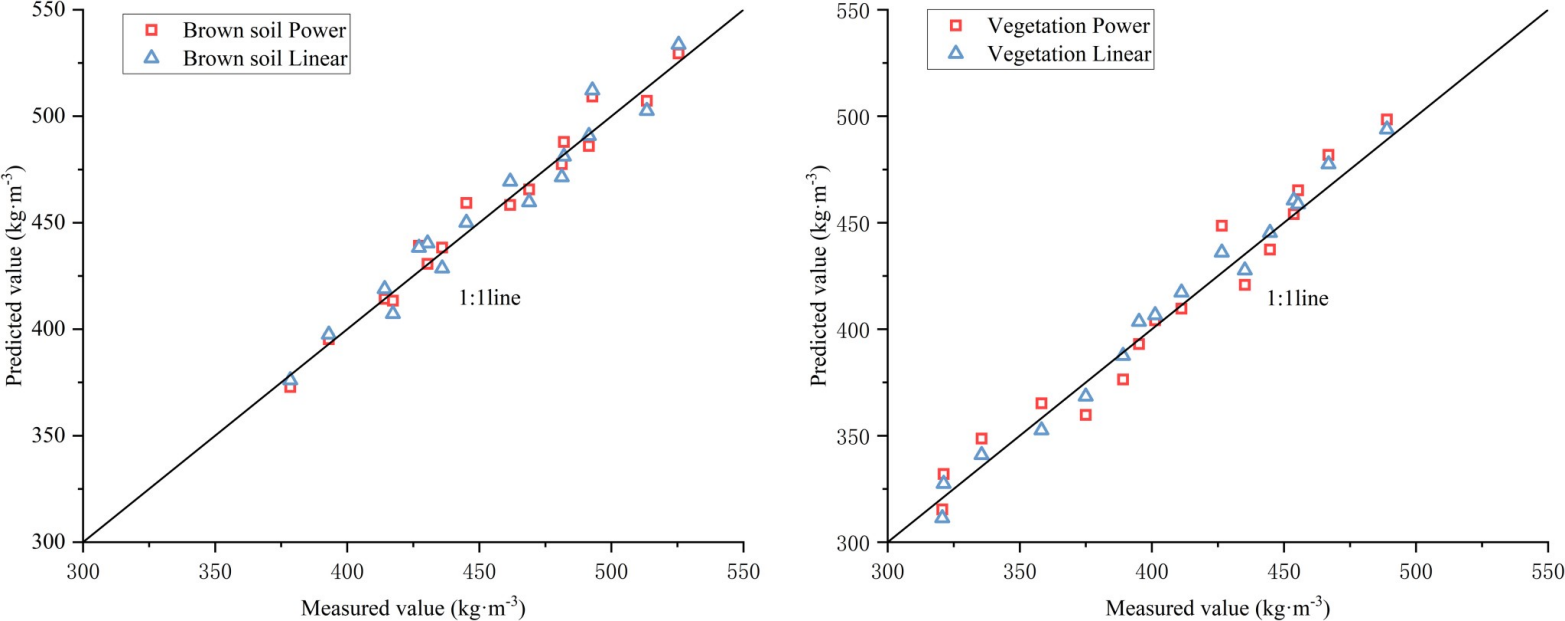
Predicted (using Table 6) and measured of *T*_*c*_.

Table 5 shows that the P-value of each variable in the two models was less than 0.01. The slope gradient and flow discharge are closely related to sediment transport capacity. The absolute values of t-test in the fitting linear function and power function model were greater than t_0.01_13 = 3.012 and F_0.01_ (2,13) = 6.701. It shows that all the models are valid. In order to further select the optimal equation, the relative error (RE), the mean relative error (MRE), the mean absolute relative error (MARE), the determination coefficient (R^2^), and the Nash-Sutcliffe model efficiency (NSE) were used to evaluate the simulated results of the equations. The results were shown in Table 6, and the measured and predicted (Eqs 3–6) values were plotted in Fig 4.

**Table 6:** Statistical results of predicted and measured sediment transport capacity (*T*_*c*_).

Underlying surface	Model	Regression equation	RE%	MRE%	MARE%	NSE	R^2^	
Brown soil slope	Linear equation	*T*_*c*_ = 10.365*θ*+214.119*Q*+270.079	-3.944–2.417	-0.272	1.664	0.954	0.957	(3)
	Power equation	*T*_*c*_ = 404.236*θ*^0.149^*Q*^0.233^	-3.342–1.488	-0.347	1.215	0.970	0.973	(4)
Vegetation slope	Linear equation	*T*_*c*_ = 14.803*θ*+166.206*Q*+212.771	-5.182–4.065	-0.473	2.335	0.953	0.959	(5)
	Power equation	*T*_*c*_ = 294.418*θ*^0.243^*Q*^0.201^	-2.286–2.911	-0.504	1.546	0.983	0.988	(6)

Table 6 shows, the model’s reliability is high due to the NSE being more generous than 0.9. Both Eq 3 (R^2^ = 0.957, NSE = 0.954) and Eq 4 (R^2^ = 0.0.973, NSE = 0.970) can predict sediment transport capacity well of brown soil slope (Fig 4). The most relative error (RE) values of Eq 3 range between -3.944% and 2.417%, with its MRE -0.272% and MARE 1.664%. The most relative error (RE) values of Eq 4 range between -3.342% and 1.488%, with its MRE -0.347% and MARE 1.215%. RE and MARE of the power function are less than those of the linear model. Both R^2^ and NSE of the power function model are slightly larger than the linear function model. It shows that the power function model is reliable. Therefore, Eq 4 is selected as the optimal formula for predicting the sediment transport capacity of the brown soil slope.

Similarly, both Eq 5 (R^2^ = 0.959, NSE = 0.953) and Eq 6 (R^2^ = 0.988, NSE = 0.983) can predict sediment transport capacity well of vegetation slope (Fig 4). The most relative error (RE) values of Eq 5 range between -5.182% and 4.065%, with its MRE -0.473% and MARE 2.335%. The most relative error (RE) values of Eq 6 range between -2.286% and 2.911%, with MRE -0.504% and MARE 1.546%. RE and MARE of the power function are less than those of the linear model. Both R^2^ and NSE of the power function model are slightly larger than that of the linear function model, and the R^2^ and NSE of Eq 6 are greater than 0.9. It shows that the power function model is reliable. Therefore, Eq 6 is selected as the optimal formula for predicting the sediment transport capacity of vegetation slope.

**Fig 5:**
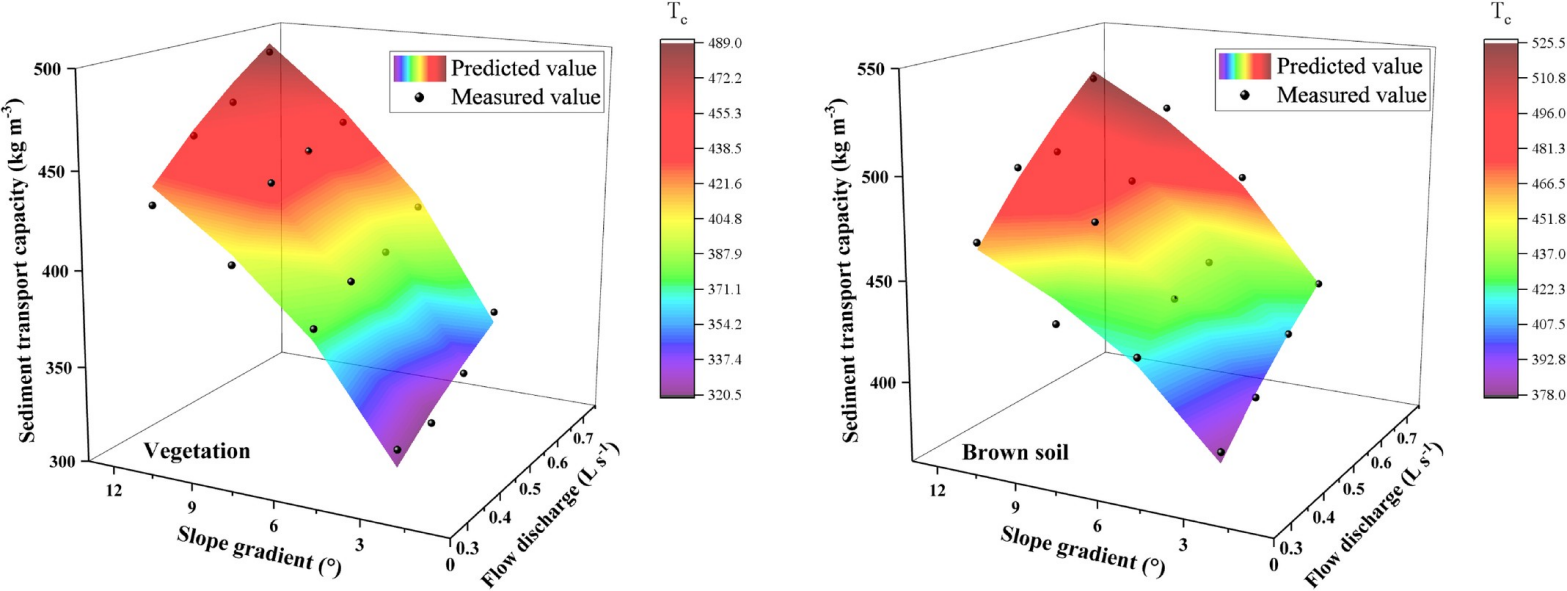
The measured values were distributed in the best predicted range.

The above results indicate that the power function (Eq 4 and Eq 6) can be used to predict sediment transport capacity accurately. Fig 5 shows the distribution of the measured values in the best predicted range. The slope gradient index (0.149) was 56.38% smaller than the flow discharge index (0.233) of Eq 4, and the flow discharge index (0.201) was 20.9% smaller than the slope gradient index (0.243) of Eq 6. This result proves that the sensitivity of sediment transport capacity to flow discharge on brown soil slope is higher than that of slope gradient, and the sensitivity of sediment transport capacity of vegetation slope to slope gradient is higher than flow discharge.

## 4. Discussion

At present, there are many research pieces on the slope’s sediment transport capacity [21–25]. Due to the different test conditions, there are some differences in the test results. In this study, the sediment transport capacity of the slope increases with slope gradient and flow discharge (Figs 3 and 5). Flow discharge is more significant than that of the slope gradient on sediment transport capacity on brown soil slope, and the effect of slope gradient is more significant than that of the flow discharge on sediment transport capacity on vegetation slope. The result is consistent with that of Lei [22] and Mu [12].

Many academicians have obtained the prediction model of sediment transport capacity [22, 23, 26–28]. The formula obtained by Lei [22], Zhang [26], Ali [23], and Wang [27] was shown in Table 7.

**Table 7:** Empirical formula for sediment transport capacity of overland flow.

	Equation		
Lei [22]	*T_c_* = −0.3109+0.01718*S*+0.12703*Q*	*T*_*c*_ is sediment transport capacity, kg(m s)^-1^; *S* is energy slope, m m^-1^; *Q* is flow discharge, L min^-1^	(7)
Zhang [26]	$T_{c}=2382.32q^{1.269}S^{1.637}d_{50}^{-0.345}$	*q* is unit flow, m^2^ s^-1^; d_50_ is median particle size, m	(8)
Ali [23]	$T_{c}=0.17\times 10^{6}\frac{Q^{1.16}}{d_{50}^{0.5}}S^{2.89}$	*Q* is flow discharge, m^3^ s^-1^; *D*_50_ is median particle size, m	(9)
Wang [27]	*T_c_* = 67.68*S*^0.98^*q*^1.20^	*S* is energy slope, m m^-1^; *q* is unit flow, m^2^ s^-1^	(10)

**Fig 6:**
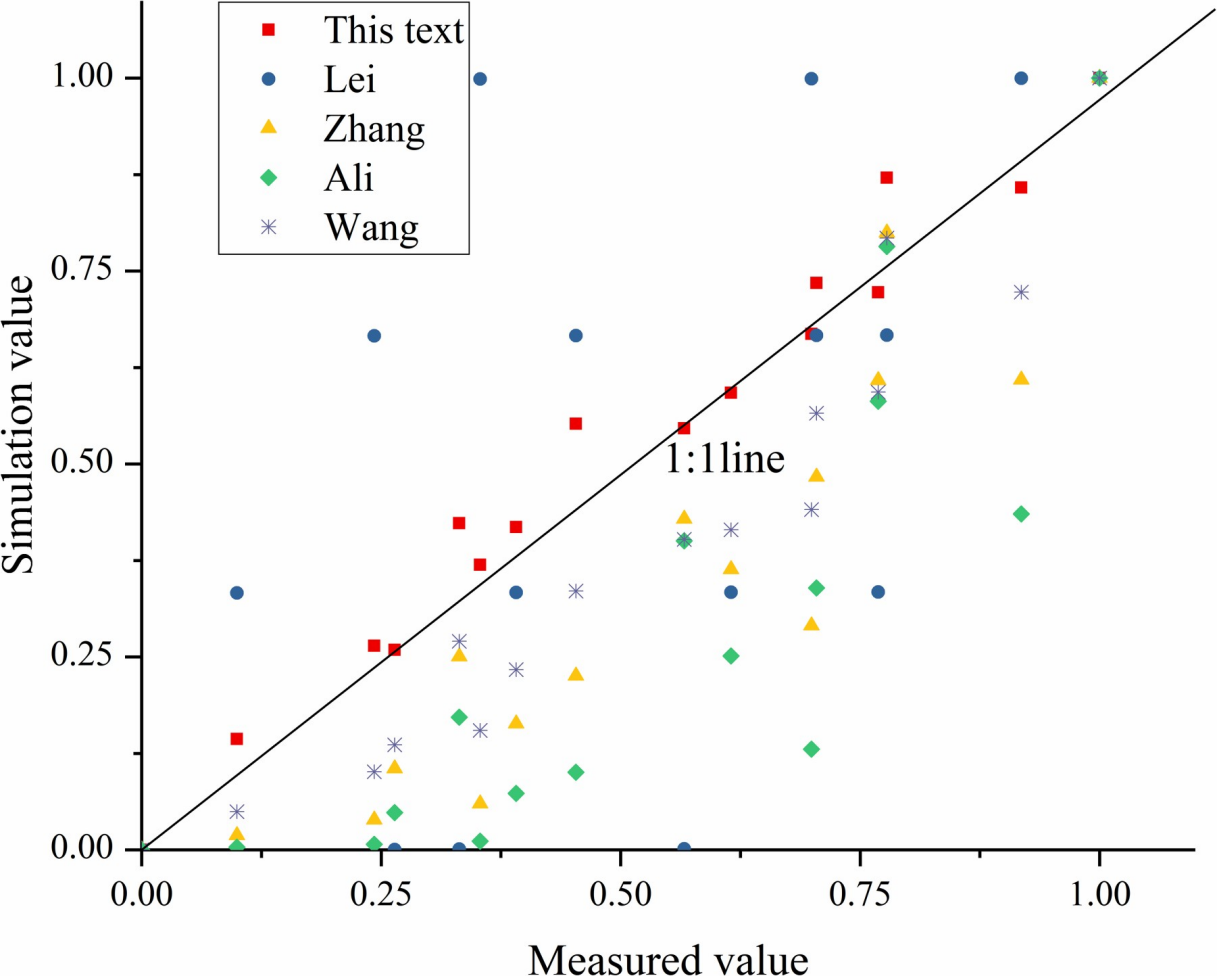
Comparison of measured and simulated values of dimensionless sediment transport capacity with different formulas.

The four scholars calculated the sediment transport capacity from the sediment transport rate concept, the concept of maximum sediment concentration is adopted in this paper, and the influence of unit flow is eliminated. Dimensionless treated for the measured value and simulated value. Its purpose is to eliminate the impact of the unit and other factors. It is drawing into the coordinate system (Fig 6).

The data points were distributed on both sides of the 1:1 line (Fig 6). The Eq (3) simulation effect is good. The simulated values calculated by other scholars’ empirical formulas are different from the measured values in this experiment. Among them, Lei [22] adopted the loess slope (< 0.01 mm, 56%), Zhang [26] adopted the bed soil slope of Yongding River in Beijing (0.1 mm < d_50_ < 1.16 mm), Ali [23] adopted coarse sand slope (0.233 mm < d_50_ < 1.022 mm), Wang [27] adopted the loess slope (0.002 mm—0.05 mm, 86.3%), and in this study adopted the brown soil slope (d_50_ = 0.29 mm, 0.05 mm—0.5 mm, 86.53%). The different types of soil selected by each scholar lead to different slope gradients and flow discharge effects on sediment transport capacity [18, 29]. However, Eq (4) is applicable to brown soil slope that the slope gradient less than 12° and flow discharge less than 0.65L·s^-1^. Therefore, the applicability of Eq (4) should be further verified for other types of larger slope gradients and flow discharges.

This study compared the sediment transport capacity change between brown soil slope and vegetation slope (Fig 3). It was found that the sediment transport capacity of the vegetation slope is 14.27% higher than that of the brown soil slope. With the increase of slope gradient and flow discharge, the increased range of sediment transport capacity of vegetation slope (46.93%) was greater than that of brown soil slope (33.98%). Different underlying surfaces lead to the change of flow velocity and the Manning coefficient and affect the sediment transport capacity [30–32]. With the increase of vegetation coverage, Reynolds number decreased [33], and Manning coefficient increased [34–36]. However, the flow state under plant cover is complex. Laminar, turbulent, subcritical, and supercritical cannot be determined [12]. The velocity is the critical factor in determining the sediment transport capacity [37]. It decreased with the increase of vegetation coverage [36], and it is the basis of hydrodynamic parameters calculation [38]. Therefore, the hydrodynamic mechanism of sediment transport capacity should be further studied for different vegetation cover.

## 5. Conclusion

Study the effects of slope gradient and flow discharge on sediment transport capacity for brown soil slope and vegetation slope. The sediment transport capacity was measured of brown soil slope and 100% canopy cover slope, and the conditions of flow discharge from 0.35 to 0.65 L·s^-1^ and slope gradient from 3 to 12°. According to the experimental study, the following conclusions can be drawn:

1. Sediment transport capacity is positively correlated with flow discharge and slope gradient. The sediment transport capacity was more sensitive to flow discharge than the slope gradient of the brown soil slope, and the sediment transport capacity was more sensitive to slope gradient than flow discharge of vegetation slope. Under the same flow discharge and slope gradient conditions, the vegetation slope’s sediment transport capacity is 11.80% higher than the brown soil slope.
2. The sediment transport capacity is closely related to the interaction of slope and discharge. The prediction equation of sediment transport capacity on brown soil slope and vegetation slope can be expressed by a power function (R^2^ = 0.982, R^2^ = 0.993).
